# Transmantle pressure under the influence of free breathing: non-invasive quantification of the aqueduct pressure gradient in healthy adults

**DOI:** 10.1186/s12987-024-00612-x

**Published:** 2025-01-03

**Authors:** Pan Liu, Kimi Owashi, Heimiri Monnier, Serge Metanbou, Cyrille Capel, Olivier Balédent

**Affiliations:** 1 Medical Image Processing Department, CHU Amiens-Picardie University Hospital, Amiens, France; 2 CHIMERE UR 7516, Jules Verne University of Picardy, Amiens, France; 3 Radiology Department, CHU Amiens-Picardie University Hospital, Amiens, France; 4 Neurosurgery Department, CHU Amiens-Picardie University Hospital, Amiens, France

**Keywords:** Transmantle pressure, Aqueduct pressure gradient, Real-time imaging, Phase contrast MRI, Breathing effects, CSF, Hydrocephalus

## Abstract

**Background:**

The pressure gradient between the ventricles and the subarachnoid space (transmantle pressure) is crucial for understanding CSF circulation and the pathogenesis of certain neurodegenerative diseases. This pressure can be approximated by the pressure difference across the aqueduct (ΔP). Currently, no dedicated platform exists for quantifying ΔP, and no research has been conducted on the impact of breathing on ΔP. This study aims to develop a post-processing platform that balances accuracy and ease of use to quantify aqueduct resistance and, in combination with real-time phase contrast MRI, quantify ΔP driven by free breathing and cardiac activities.

**Methods:**

Thirty-four healthy participants underwent 3D balanced fast field echo (BFFE) sequence and real-time phase contrast (RT-PC) imaging on a 3T scanner. We used the developed post-processing platform to analyse the BFFE images to quantify the aqueduct morphological parameters such as resistance. RT-PC data were then processed to quantify peak flow rates driven by cardiac and free breathing activity (Qc and Qb) in both directions. By multiplying these Q by resistance, ΔP driven by cardiac and breathing activity was obtained (ΔPc and ΔPb). The relationships between aqueduct resistance and flow rates and ΔP driven by cardiac and breathing activity were analysed, including a sex difference analysis.

**Results:**

The aqueduct resistance was 78 ± 51 mPa·s/mm³. The peak-to-peak cardiac-driven ΔP (Sum of ΔPc^+^ and ΔPc^−^) was 24.2 ± 11.4 Pa, i.e., 0.18 ± 0.09 mmHg. The peak-to-peak breath-driven ΔP was 19 ± 14.4 Pa, i.e., 0.14 ± 0.11 mmHg. Males had a longer aqueduct than females (17.9 ± 3.1 mm vs. 15 ± 2.5 mm, *p* < 0.01) and a larger average diameter (2.0 ± 0.2 mm vs. 1.8 ± 0.3 mm, *p* = 0.024), but there was no gender difference in resistance values (*p* = 0.25). Aqueduct resistance was negatively correlated with stroke volume and the peak cardiac-driven flow (*p* < 0.05); however, there was no correlation between aqueduct resistance and breath-driven peak flow rate.

**Conclusions:**

The highly automated post-processing software developed in this study effectively balances ease of use and accuracy for quantifying aqueduct resistance, providing technical support for future research on cerebral circulation physiology and the exploration of new clinical diagnostic methods. By integrating real-time phase contrast MRI, this study is the first to quantify the aqueduct pressure difference under the influence of free breathing. This provides an important physiological reference for further studies on the impact of breathing on transmantle pressure and cerebral circulation mechanisms.

**Supplementary Information:**

The online version contains supplementary material available at 10.1186/s12987-024-00612-x.

## Background

Cerebrospinal fluid (CSF) is essential for the central nervous system, providing protection, maintaining intracranial pressure stability, and facilitating metabolic waste removal [1–3]. Abnormal CSF circulation is linked to several neurodegenerative diseases [4–9]. Transmantle pressure, the pressure gradient between the ventricles and the subarachnoid space (SAS), is a critical parameter in CSF circulation. Accurate quantification of transmantle pressure is vital for better understanding CSF circulation mechanisms and the pathogenesis of neurodegenerative diseases such as normal pressure hydrocephalus and Chiari malformations [10–12]. Additionally, it may influence the CSF hydrodynamics within the perivascular space, thereby affecting the efficiency of waste clearance in the brain [1, 13, 14].

Traditionally, transmantle pressure has been measured directly using pressure sensors placed between the subarachnoid space and the ventricles. However, accurately quantifying pressure variations at such low levels is challenging for sensors [4, 15, 16], as transmantle pressure typically ranges from 0.1 to 0.4 mmHg, i.e. 13 to 53 Pa [15, 17], compared to intracranial pressure amplitudes that reach around 2 mmHg [18, 19]. Additionally, the invasive nature of this measurement technique increases the risk of infection, further limiting its clinical applicability.

Non-invasive quantification of transmantle pressure can be achieved by integrating morphological assessments with measurements of aqueduct flow, significantly enhancing clinical applicability. The aqueduct, a slender and elongated channel linking the third and fourth ventricles, is the primary site of transmantle pressure drop. Therefore, the pressure difference (ΔP) between the ends of the aqueduct can indirectly reflect transmantle pressure.

Currently, there are two primary approaches to calculating ΔP. The first approach involves high-precision morphological modelling based on morphological images, followed by ΔP calculations using specialized fluid dynamics simulation software. This method has a complex post-processing workflow, requires extensive prior knowledge, and is highly dependent on boundary conditions such as flow and geometry, and involves substantial computational time, making it more suitable for research purposes and limiting its clinical applicability [20–22]. The second approach employs a simplified mathematical model and an approximation of the aqueduct diameter to calculate ΔP [17]. However, the accuracy of this method is challenged by the significant impact of morphological changes in the aqueduct on ΔP. While this approach can significantly reduce post-processing complexity and provide an approximate range of ΔP, the lack of finite element segmentation of the aqueduct may limit the accuracy of ΔP quantification.

To enhance clinical applicability, it is important to balance accuracy and efficiency when calculating ΔP. To our knowledge, there is currently no tool that can efficiently and quickly perform finite element segmentation to calculate ΔP. Additionally, recent studies using real-time phase contrast MRI (RT-PC) have shown that breathing significantly impacts CSF dynamics [27–30]. However, there is a lack of research quantifying the effect of breathing on ΔP.

To address these challenges, we developed a highly automated platform that performs finite element segmentation of aqueduct morphology and rapid calculation of ΔP using flow rate data. The aim of this study was to utilize this platform with continuous flow rate data obtained from real-time phase contrast MRI (RT-PC) to quantify ΔP under the influences of cardiac and free breathing activities.

## Methods

### Participants

This study was approved by the local investigational review board (CPP Nord Ouest II, Amiens, France; reference: PI2019_843_0056) and was performed in accordance with the Declaration of Helsinki.

The study comprised 34 healthy adults, including 16 females and 18 males, with a mean age of 25 ± 4 years (range 19–35 years). The examinations lasted approximately 30 min. Prior to participation, all individuals received a detailed explanation of the study’s objectives and methods and provided written informed consent. Exclusion criteria included contraindications to MRI and a history of cerebrovascular or respiratory diseases.

### Theoretical basis for ΔP calculation

The aqueduct has a slight curvature and an approximately circular cross-Sects. [31, 32]. The Reynolds number is well below 2000, and the Womersley number is typically less than 5, indicating laminar flow dominated by viscous forces [24]. Therefore, we approximate the aqueduct as a straight tube with a varying diameter along its length. Poiseuille’s formula is used to calculate ΔP by summing the contributions of individual cross-sections along the aqueduct, involving two key components: resistance (R) calculated from morphological images and flow (Q) obtained from RT-PC images.

$$\:\varDelta\:P=R \cdot \:Q$$

where R is given by:

$$\:R=\:\frac{128\cdot\:\mu\:\cdot\:L}{\pi\:\cdot\:{D}^{4}}$$

Where µ represents the dynamic viscosity and has been set to 0.71 mPa·s in this study (µ of water at 36 °C), L denotes the length, and D represents the diameter of the aqueduct element.

### Image acquisition

Images were acquired using a 3T MRI scanner (Philips Achieva; maximum gradient = 80 mT/m; slew rate = 120 mT/m/ms) equipped with a 32-channel head coil, with participants in the supine position.

**Fig. 1:**
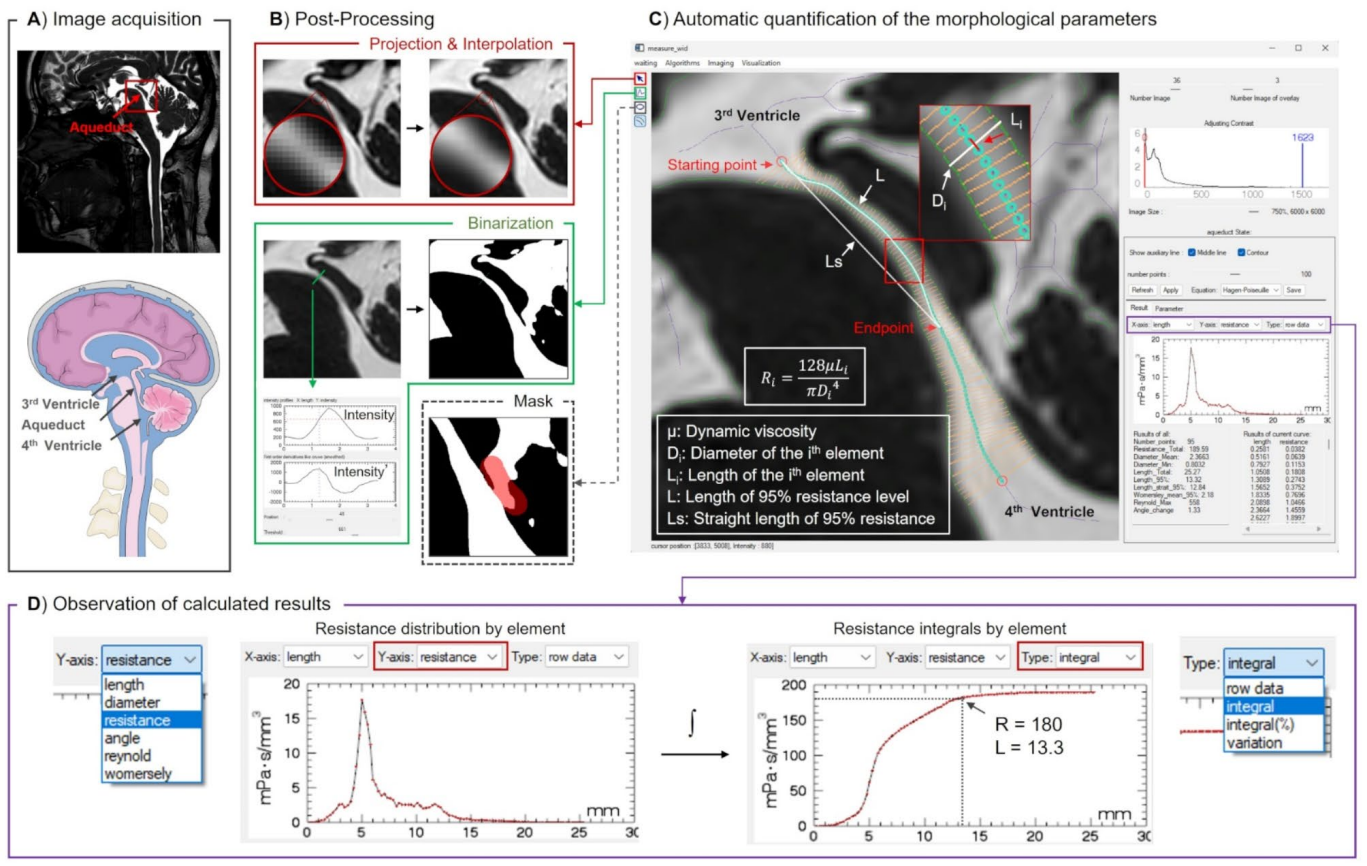
(**A**) BFFE morphological images. (**B**) Post-processing for cerebral aqueduct resistance quantification. Spatial resolution enhancement, automatic image binarization and mask inclusion. (**C**) The software automatically performs a finite element analysis of the aqueduct to derive the resistance of each element. Parameters such as resistance, mean diameter and length were extracted. (**D**) The resistance distribution (left) and integral (right) profiles are shown. The endpoint is defined at 95% of the total resistance, corresponding in this example to a resistance (R) of 180 mPa·s/mm^3^ and a length (L) of 13.3 mm

A sagittal 3D Balanced Fast Field Echo (BFFE) sequence was used for morphological imaging (Fig. 1-A). This sequence produces high-resolution images and differentiates between CSF and adjacent brain tissue, allowing for a detailed examination of CSF pathways. The parameters of BFFE sequence were as follows: repetition time (TR) = 5.5 ms, echo time (TE) = 2.2 ms, field of view (FOV) = 180 × 180 × 90 mm^3^, spatial resolution of acquisition = 0.6 × 0.6 × 1.2 mm^3^, number of images = 75, flip angle = 45°, acquisition matrix: 300 × 300, acquisition time = 182 s, and sensitivity encoding factor = 1.5.

**Fig. 2:**
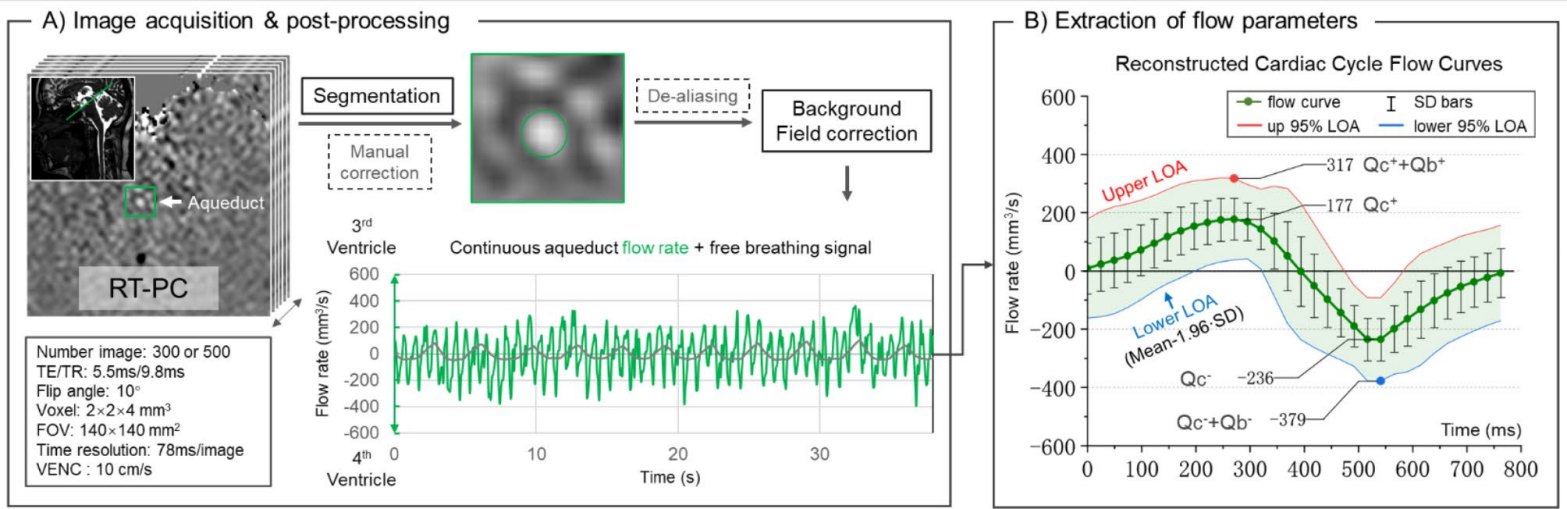
(**A**) RT-PC MRI imaging levels and parameters. Continuous flow signal was obtained after post-processing. (**B**) The continuous flow signal was reconstructed as an average flow curve with 95% limits of agreement (LOA). Qc^+^ represents the maximum cardiac-driven flow rate (4th toward the 3rd ventricle), and Qc^+^ + Qb^+^ represents the maximum cardiac- and breath-driven flow rate

RT-PC was used to measure the CSF flow rate through the aqueduct of Sylvius (Fig. 2-A). RT-PC is based on echo planar imaging sequence with a Cartesian trajectory, each phase-contrast image is reconstructed by the subtraction of two velocity images: a flow-encoded image and a flow-compensated image. The positive direction is defined as from the 4th ventricle towards the 3rd ventricle. The acquisition plane was positioned perpendicular to the aqueduct using BFFE images as a reference. The parameters of RT-PC sequence were as follows: TR = 10.2 ms, TE = 6 ms, FOV = 140 × 140 mm^2^, acquisition spatial resolution = 2 × 2 mm^2^, slice thickness = 4 mm, number of images = 300 or 500, imaging speed = 87 ms/image, flip angle = 10°, velocity encoding = 10 cm/s, EPI-factor = 7 (indicating that 7 echoes were collected during each TR), sensitivity encoding factor = 2.5. During the acquisition process, all participants maintained free breathing, and this study did not require the use of sensors to record breathing signals.

### Morphological images post-processing: calculation of the resistance − R

A highly automated post-processing platform has been developed using the IDL (Interactive Data Language) programming language for the quantification of aqueduct resistance [33]. The post-processing workflow for BFFE images is as follows:

1. 2–3 BFFE images were selected for maximum intensity projection to obtain the aqueduct projection, and then linear interpolation was applied to increase the spatial resolution to 0.03 × 0.03mm^2^ (Fig. 1-B).
2. A line was drawn at the narrowest point of the aqueduct. The software identified the maximum intensity gradient in the pixel intensity profile and used the corresponding pixel intensity as the binarization threshold.
3. The platform allows for manual application of masks to exclude cerebellar CSF, thereby isolating the aqueduct to avoid errors in subsequent edge detection, centreline definition, and finite element segmentation.
4. By manually defining the aqueduct starting point and endpoint, the platform automatically delineates the aqueduct boundary and centreline, dividing it into multiple elements (defaulting to 100). Parameters such as length, diameter, angle, and the corresponding value of resistance are calculated for each element (Fig. 1-C).
5. Results can be directly observed and copied within the platform or can also be saved to a .txt file (Fig. 1-D).

As shown in Fig. 1-C, the main resistance contribution is located at the narrowing of the aqueduct, while the resistance value at the terminal end (entrance to the 4th ventricle) is very low. To minimize inter-operator variation in measuring the length of the aqueduct, the software automatically identifies the 95th percentile of the total resistance value as the final endpoint. All subsequent parameter calculations are confined to the section between the starting point and this endpoint.

The following results were obtained from the BFFE images:

- R: Total resistance from the starting point to the endpoint of the aqueduct.
- L: Path length of the aqueduct (starting point to endpoint).
- Ls: Straight-line distance of the aqueduct (starting point to endpoint).
- L-defined: The operator-defined path length (starting point to defined endpoint).
- D-mean: Average diameter of the aqueduct (starting point to endpoint).
- D-min: Minimum diameter across all aqueduct elements.
- Womersley-mean: Average Womersley number across aqueduct elements.
- Reynold-max: Maximum Reynolds number across aqueduct elements.

Womersley Number is calculated as:

$$\:Womersley\:number=\:\frac{D}{2} \cdot \:\sqrt{\frac{\omega\: \cdot \:\rho\:}{\mu\:}}$$

And, Reynolds Number is calculated as:

$$\:Reynolds\:number=\:\frac{\rho\: \cdot \:v \cdot \:D}{\mu\:}$$

Where D represents the diameter of the aqueduct element, ω represents the angular frequency of the pulsatile CSF flow (obtained by multiplying the heart rate by 2π), µ represents the dynamic viscosity of the CSF (0.71 mPa·s), ρ represents the density of the CSF (1000 kg/m^3^), and v represents the flow velocity.

### RT-PC images post-processing: quantification of the flow rate − Q

Previous research has confirmed the accuracy of RT-PC in quantifying aqueduct CSF flow [28]. In this study, we used Flow 2.0 software, developed in IDL, for RT-PC sequence post-processing [34]. The post-processing workflow for RT-PC images is as follows (Fig. 2 & Fig. S6):

1. The aqueduct’s ROI is determined by analysing the frequency domain characteristics of pixel velocity curves, with an emphasis on the proportion of the cardiac frequency component [35]. The software supports manual correction of the ROI.
2. Extract the continuous flow (rate) curve within the ROI, for the velocities that exceed the VENC can be corrected by the de-aliasing function.
3. The software automatically detects stationary tissue regions surrounding the aqueduct in the RT-PC images. The average velocity of this region is used as the new zero velocity point to complete background correction.
4. The software identifies the minimum flow rate point of each cardiac cycle by analysing the sampling frequency and heart rate. These points are then used as segmentation points to divide the continuous flow signal, which contains several complete breathing cycles, into multiple independent flow curves.
5. Cubic spline interpolation is used to increase the number of sampling points for each independent flow curve to 32. These independent flow curves are then combined to reconstruct a new flow curve with standard deviation (SD). The 95% limits of agreement (Upper/Lower LOA) are calculated as the mean ± 1.96 × SD (Fig. 2-B).

A previous study [28] has demonstrated that the reconstructed flow curve closely aligns with the flow curve obtained from conventional cardiac-gated phase-contrast imaging and can be considered as representing the mean cardiac-driven flow. Additionally, breathing significantly affects the net flow rate and the amplitude of each cardiac cycle, which can be approximated as vertical shifts or scaling in the reconstructed flow curve [36]. Therefore, the extremes of the reconstructed flow curve indicate cardiac-driven flow (Qc+ & Qc-), while the differences between the extremes of LOA and the extremes of the reconstructed flow curve indicate breathing-driven flow (Qb+ & Qb-). In this study, we used the 95% LOA to reduce the influence of ultra-low-frequency fluctuations and noise, thus more accurately representing the flow variations driven by breathing. The mean cardiac-driven flow (Qc) is calculated as the average of Qc + and Qc-, and similarly, the mean breathing-driven flow (Qb) is determined (Fig. 2-B).

The following results were obtained from the RT-PC images:

- Qc^+^: The maximum cardiac-driven flow rate, from 4th to 3rd ventricle.
- Qc^-^: The maximum cardiac-driven flow rate, from 3rd to 4th ventricle.
- Qc: The average of Qc^+^ and Qc^−^, it can be considered as half of the flow rate amplitude.
- Qb^+^: The maximum (free) breath-driven flow rate, from 4th to 3rd ventricle.
- Qb^-^: The maximum breath-driven flow rate, from 3rd to 4th ventricle.
- Qb: The average of Qb^+^ and Qb^−^.
- D-PC: The diameter of the aqueduct measured by RT-PC.
- Q-net: The net flow rate of the reconstructed flow curve.
- SV: The average of positive and negative stroke volume. It represents the volume of fluid oscillating within the averaged cardiac cycle through the cross-section.

Furthermore, the cross-section includes two internal carotid arteries and the basilar artery. Nevertheless, due to the low velocity encoding, aliasing occurs in the centre of these arteries. Conversely, the edges of the vessels with lower flow velocities do not exhibit aliasing, which facilitates the identification of the systolic and diastolic phases of the heart. The cardiac systolic and diastolic phases were defined by extracting the flow curves at the boundaries of the internal carotid artery, and subsequently, the heart-driven CSF fluid dynamics were analysed.

### Calculation of cardiac-driven and breath-driven ΔP

By multiplying the aqueduct’s resistance (R) with the cardiac-driven and breath-driven flow rates (Qc and Qb), we obtain the following pressure differences of aqueduct (ΔP):

- ΔPc+: The maximum cardiac-driven ΔP, from 4th to 3rd ventricle.
- ΔPc- : The maximum cardiac-driven ΔP, from 3rd to 4th ventricle.
- ΔPc: The average of ΔPc + and ΔPc-.
- ΔPb+: The maximum breath-driven ΔP, from 4th to 3rd ventricle.
- ΔPb- : The maximum breath-driven ΔP, from 3rd to 4th ventricle.
- ΔPb: The average of ΔPb + and ΔPb-.

### Statistical analysis

Statistical analyses were performed using R software. Data are presented as mean ± standard deviation (SD), range (minimum-maximum), and coefficient of variation (CV). Differences between groups were analysed using the Wilcoxon test: the Wilcoxon signed-rank test for paired data and the Wilcoxon rank-sum test for unpaired data. Correlations between groups were assessed using the Spearman test. All tests were two-tailed, with significance set at *p* < 0.05. For the comparison of multiple parameters, independent significance or correlation tests were conducted for each parameter. Given the exploratory nature of the study, no corrections for multiple comparisons were applied. This increases the risk of Type I errors, and results should therefore be interpreted with caution.

## Results

The preliminary results of this study and the software demonstration have been presented at a conference [36].

BFFE and RT-PC imaging were successfully completed for all participants, comprising 18 males with an average age of 26 ± 4 years (range 20–35 years) and 16 females with an average age of 25 ± 3 years (range 19–32 years). There was no statistical difference in age between males and females.

### Aqueduct morphological parameters and resistance

**Fig. 3:**
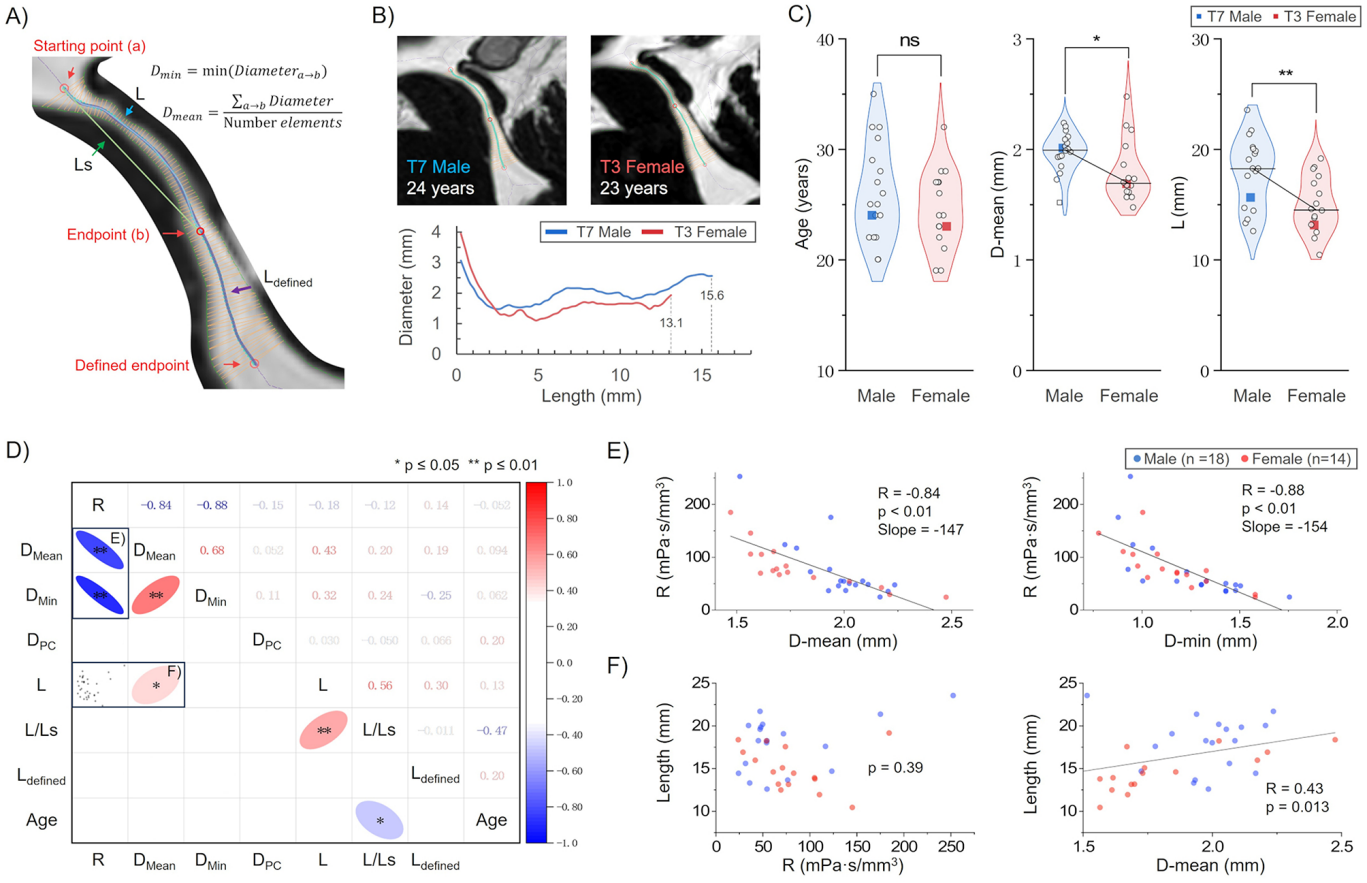
(**A**) Diagram illustrating morphological parameters measured within the aqueduct. (**B**) Segmentation examples of the aqueduct from a 24-year-old male (T7) and a 23-year-old female (T3) participant, including curves of their aqueduct radius changes along the length. (**C**) Distribution of age, average aqueduct radius (D-mean), and aqueduct length (L) for male (blue) and female (red) participants, with blue and red squares representing T7 and T3 participants, respectively. (**D**) Correlation matrix of morphological parameters with Spearman’s correlation test, the upper triangle of the matrix represents the r values, while the lower triangle shows correlation ellipses and significance levels, with * indicating *p* < 0.05 and ** indicating *p* < 0.01; the color bar corresponds to the r values. R for resistance and DPC for diameter measured by RT-PC. (**E**) Scatter plots depicting the relationships between R and D-mean and between R and D-min. (**F**) Scatter plots depicting the relationship between length and R and between length and D-mean

The morphological parameters of the aqueduct are shown in Table 1, and a visual representation of these parameters is given in Fig. 3-A. The average total resistance (R) of the population was 78 ± 51 mPa·s/mm³, with the highest CV at 64%. The aqueduct elements had an average Womersley number ranging from 2.2 to 5.0; the maximum Reynolds number was less than 504. The L/Ls ratio varied between 1.03 and 1.08. The diameter obtained from RT-PC (D-PC) was significantly larger than the mean diameter (D-mean) obtained from BFFE (*p* < 0.01, Z = 2.92, *r* = 0.50), with values of 3.0 ± 0.4 mm and 1.9 ± 0.2 mm, respectively. Furthermore, there was a noticeable difference in inter-individual variability between the L-defined and the L, with a CV of 9% and 19%, respectively.

**Table 1:** Quantitative results of morphological parameters of the aqueduct. SD is the standard deviation and CV is the coefficient of variation. D-Mean and D-min refer to the mean and minimum diameter of the aqueduct from starting point to endpoint of aqueduct. D-PC indicates the diameter measured in RT-PC images. L and ls are the path length and the direct linear distance from the starting point to the endpoint, L-defined indicates the path length of the starting point to the initial defined endpoint. Statistical differences between male and female were determined using the wilcoxon rank-sum test, with * indicating *p* < 0.05 and ** indicating *p* < 0.01, Z-value indicating the deviation of the observed rank-sum from its expected value under the null hypothesis, the effect size r is calculated as the Z value divided by the square root of the total sample size (*n* = 34)

	Resistance	D-mean	D-min	D-PC	L	Ls	L/Ls	L-defined	Womersley_mean_	Reynold_max_
Unit	(mPa·s/mm^3^)	(mm)	(mm)	(mm)	(mm)	(mm)	-	(mm)	-	-
**All** (*n* = 34)										
Mean ± SD	78 ± 51	1.9 ± 0.2	1.2 ± 0.2	3.0 ± 0.4	16.5 ± 3.2	15.7 ± 2.9	1.05 ± 0.01	27 ± 2.4	3.1 ± 0.5	251 ± 95
Range	24 − 253	1.5 − 2.5	0.8 − 1.8	2.0 − 3.6	10.4 − 23.6	10.1 − 22	1.03 − 1.08	23 − 31	2.2 − 5.0	98 − 504
CV	64%	13%	19%	14%	19%	19%	1.1%	9%	15%	38%
**Male** (*n* = 18)										
Mean ± SD	75 ± 57	2.0 ± 0.2	1.2 ± 0.2	3.1 ± 0.3	17.9 ± 3.1	17 ± 2.8	1.05 ± 0.01	28 ± 2.1	3.1 ± 0.3	267 ± 95
Range	24 − 253	1.5 − 2.2	0.8 − 1.8	2.6 − 3.6	12.6 − 23.4	12.2 − 22	1.03 − 1.08	24 − 31	2.2 − 3.5	108 − 504
CV	76%	9%	19%	10%	17%	16%	1.2%	7.4%	11%	36%
**Female** (*n* = 16)										
Mean ± SD	81 ± 40	1.8 ± 0.3	1.2 ± 0.2	2.9 ± 0.5	15 ± 2.5	14.2 ± 2.3	1.05 ± 0.01	26 ± 1.8	3.0 ± 0.6	233 ± 92
Range	24 − 184	1.5 − 2.2	0.8 − 1.6	2.0 − 3.6	10.4 − 19.2	10.1 − 18.2	1.03 − 1.07	23 − 30	2.3 − 5	98 − 468
CV	50%	15%	19%	17%	17%	16%	0.9%	7.2%	19%	39%
**Male vs. Female**										
p (Wilcoxon)	0.25	0.024*	0.32	0.57	0.009**	0.007**	0.72	0.004**	0.09	0.31
U value		210			220	223		229		
Z value		2.26			2.61	2.71		2.92		
Effect size (r)		0.39			0.46	0.48		0.52		

A comparison of the morphological parameters of males and females (as shown in Table 1; Fig. 3-C) revealed that the average diameter of the aqueduct in male was significantly greater than that in female (2.0 ± 0.2 mm vs. 1.8 ± 0.3 mm, *p* = 0.024, *r* = 0.39). Furthermore, the length of the aqueduct in male was also significantly greater than that in female (17.9 ± 3.1 mm vs. 15 ± 2.5 mm, *p* < 0.01, *r* = 0.46). There was no significant difference in resistance between the sexes. However, the coefficient of variation of resistance was found to be higher in male (76%) than in female (50%).

Fig. 3-D presents a correlation matrix between various morphological parameters. This analysis reveals a significant positive correlation between resistance and both the average diameter and the minimum diameter of the aqueduct, with the correlation coefficient of -0.84 and − 0.88, respectively (Fig. 3-E). Resistance does not correlate with the length of aqueduct.

### Aqueduct flow parameters and pressure gradient

**Fig. 4:**
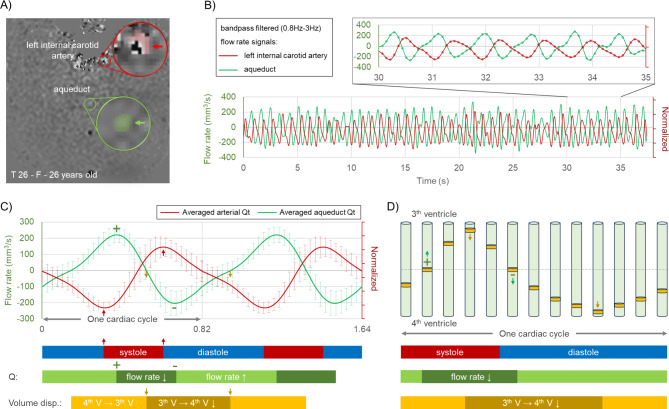
(**A**) Intracranial sectional RT-PC image of participant T26, delineating the left internal carotid (red) and aqueduct (green) ROIs, with magnified views of the circled areas, with the surrounding low-velocity region defined as the arterial ROI due to arterial aliasing. (**B**) Flow velocity signals from the arterial and aqueduct ROIs, band-pass filtered to remove low-frequency components and noise. (**C**) Reconstructed average arterial flow curve and aqueduct flow curve. The arterial flow curve is used to distinguish the cardiac phases of systole and diastole, while the aqueduct flow curve indicates flow variations and volume displacement during these phases. (**D**) An illustration of aqueduct volume displacement over a cardiac cycle is also provided

Fig. 4 shows the CSF flow curves for the aqueduct and the blood flow curve from the inner wall of the left internal carotid artery of participant T26. The arterial flow rate curve distinguishes the phases of systole and diastole. The CSF flow rate reaches a peak during the early phase of systole, indicating a maximal velocity of CSF towards the 3rd ventricle. This is followed by a rapid decline (Fig. 4-C&D dark green). CSF flows out of the 3th ventricle from mid-systole to mid-diastole (Fig. 4-C&D dark yellow). The duration of decline in CSF flow rate aligns with the systolic phase, and the duration of CSF outflow and inflow to the 3th ventricle is approximately equal.

**Fig. 5:**
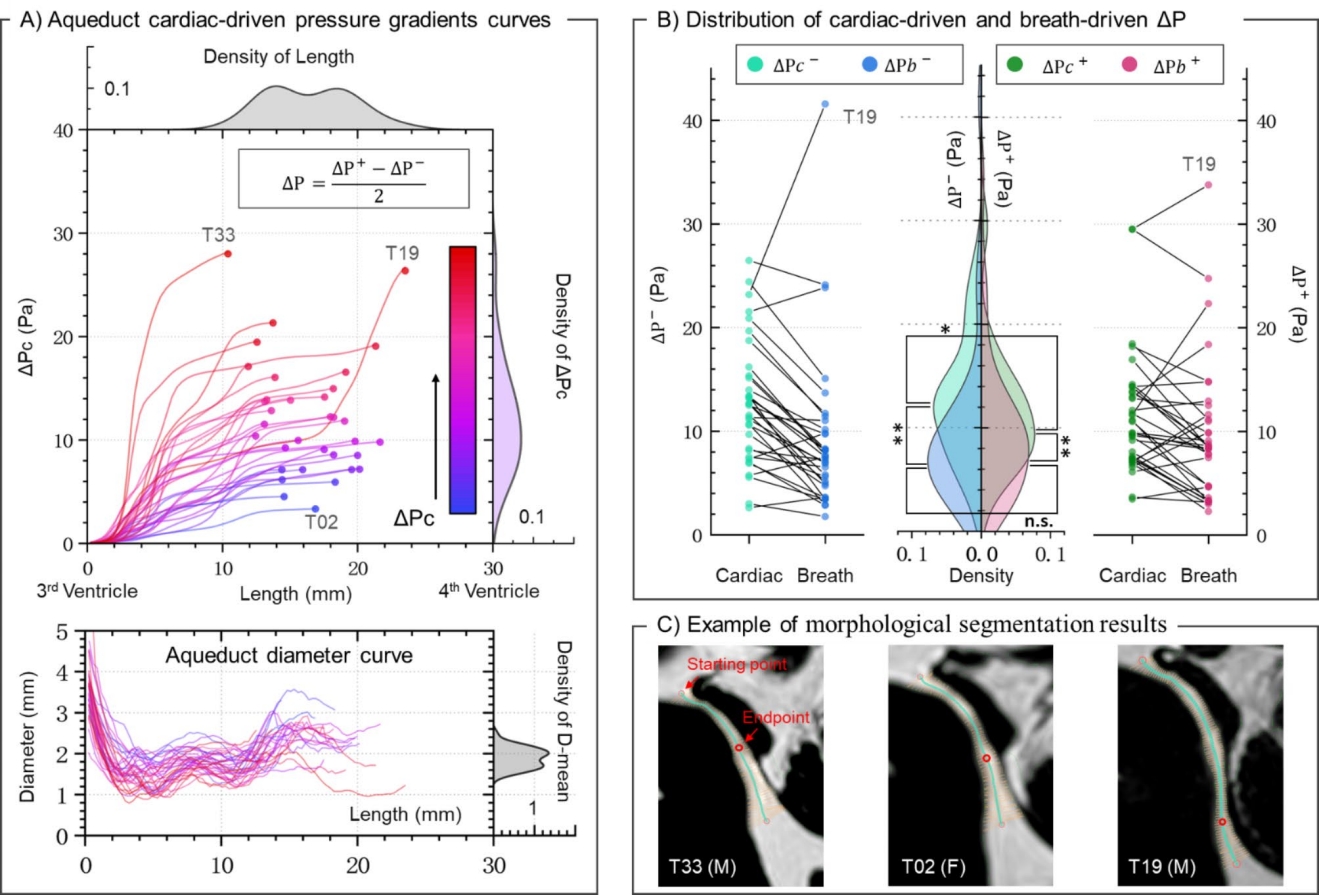
(**A**) The upper panel displays the gradient curve of cardiac-driven aqueduct pressure difference (ΔP) along the length of the aqueduct for all participants (*n* = 34), accompanied by the density distribution of ΔP and length at the endpoint. The lower panel presents the curve of aqueduct diameter variation with length, accompanied by the density distribution of the mean diameter. (**B**) This panel displays a line-connected scatter plot and the density distribution of cardiac-driven and breath-driven ΔP. The symbol “+” indicates the direction from 4th to 3rd ventricle and “-” indicates the direction from 3rd to 4th ventricle. Statistical differences between groups were determined using the Wilcoxon test, with * indicating *p* < 0.05 and ** indicating *p* < 0.01. (**C**) Morphological segmentation results of the aqueduct for three specific participants

Table 2 presents the cardiac-driven and breath-driven components of both flow rate (Qc and Qb) and pressure difference (ΔPc and ΔPb). When considering both male and female subjects collectively, the cardiac-driven Qc and ΔPc in the “-” direction (from the 3rd to the 4th ventricle) are significantly higher than those in the “+” direction (from the 4th to the 3rd ventricle), with *p*-values of 0.035 and 0.028 and effect sizes (r) of 0.36 and 0.38, respectively. In contrast, the breath-driven Qb and ΔPb show no significant differences between the two directions (Fig. 5-B).

It is notable that when considering gender differences, female subjects exhibit a significant difference in breath-driven Q and ΔP between the two directions: Qb^+^ is greater than Qb^−^ (*p* = 0.002, *r* = 0.54), and ΔPb^+^ is greater than ΔPb^−^ (*p* = 0.004, *r* = 0.49). This indicates that the breath-driven flow rates and pressure differences are greater in the direction from the 4th to the 3rd ventricle in females.

**Table 2:** Quantification of aqueduct flow and pressure difference. The symbol “+” indicates the direction from 4th to 3rd ventricle. (Breath/Cardiac) % indicates the percentage of breath-driven flow/pressure relative to cardiac-driven flow/pressure. SV represents stroke volume, and Q-net represents net flow. The wilcoxon signed-rank test was used to detect significant differences in flow or pressure between the two directions. Statistical differences between male and female were determined using the wilcoxon rank-sum test, with * indicating *p* < 0.05 and ** indicating *p* < 0.01, Z-value indicating the deviation of the observed rank-sum from its expected value under the null hypothesis, the effect size r is calculated as the Z value divided by the square root of the total sample size (*n* = 34)

	Period	Q^+^	Q^−^	+ vs. -	Q	SV	Q-net	ΔP^+^	ΔP^−^	+ vs. -	ΔP
Unit	(second)	(mm^3^/s)	(mm^3^/s)	(*p* value)	(mm^3^/s)	(mm^3^)	(mm^3^/s)	(Pa)	(Pa)	(*p* value)	(Pa)
**Cardiac-driven**	Tc	Qc^+^	Qc^−^		Qc			ΔPc^+^	ΔPc^−^		ΔPc
**All** (*n* = 34)											
Mean ± SD	0.85 ± 0.14	169 ± 72	186 ± 76	0.035*	177 ± 70	39 ± 19	0.7 ± 12	11.5 ± 6.0	12.7 ± 5.8	0.028*	12.1 ± 5.7
Range	0.54 − 1.23	56 − 402	69 − 366	*r* = 0.36	73 − 384	12 − 92	-21 − 31	3.4 − 29	2.6 − 26	*r* = 0.38	3.3 − 28
**Male** (*n* = 18)											
Mean ± SD	0.89 ± 0.15	189 ± 81	208 ± 86	0.14	199 ± 80	45 ± 23	0.8 ± 14	11.5 ± 5.6	12.3 ± 4.5	0.12	11.9 ± 4.9
Range	0.66 − 1.23	56 − 402	91 − 366		74 − 384	14 − 92	-21 − 31	6.6 − 29	6.9 − 23		7.1 − 26
**Female** (*n* = 16)											
Mean ± SD	0.80 ± 0.11	147 ± 54	161 ± 62	0.12	154 ± 54	33 ± 13	0.6 ± 10	11.4 ± 6.6	13.2 ± 7.5	0.12	12.3 ± 6.8
Range	0.54 − 1.0	56 − 254	69 − 300		73 − 277	12 − 65	-15 − 17	3.4 − 29	2.6 − 26		3.3 − 28
**Male vs. Female**											
*P* value	0.08	0.15	0.09		0.10	0.14	0.85	0.90	0.72		0.90
**Breath-driven**	Tb	Qb^+^	Qb^−^		Qb			ΔPb^+^	ΔPb^−^		ΔPb
**All** (*n* = 34)											
Mean ± SD	4.1 ± 0.9	127 ± 48	119 ± 46	0.09	124 ± 45			9.7 ± 6.9	9.3 ± 7.8	0.09	9.5 ± 7.2
Range	2.7 − 6.4	47 − 235	55 − 227		53 − 231			2.3 − 34	1.7 − 42		2.4 − 38
**Male** (*n* = 18)											
Mean ± SD	4.3 ± 1.0	122 ± 57	124 ± 54	0.6	123 ± 53			8.6 ± 7.4	8.9 ± 8.7	0.6	8.8 ± 8.0
Range	2.8 − 6.4	47 − 235	59 − 227		53 − 231			2.3 − 34	2.8 − 42		2.5 − 38
**Female** (*n* = 16)											
Mean ± SD	3.9 ± 0.8	135 ± 37	113 ± 36	0.002**	124 ± 35			10.8 ± 6.3	9.5 ± 6.6	0.004**	10.2 ± 6.4
Range	2.7 − 6	75 − 194	55 − 172	*r* = 0.54	65 − 174			3.1 − 25	1.7 − 24	*r* = 0.49	2.4 − 24
**Male vs. Female**											
*P* value	0.29	0.34	0.74		0.64			0.21	0.55		0.39

The upper image in Fig. 5-A illustrates the cardiac-driven pressure gradient curve, describing the aqueduct pressure difference from the starting point along its length. The lower image shows the variation in aqueduct diameter along its length. The density plot indicates that the distribution of the mean diameter is more compact compared to ΔPc. Figure 5-C displays the aqueduct morphology and segmentation results for three specific participants.

### The resistance, flow and pressure difference of the aqueduct

**Fig. 6:**
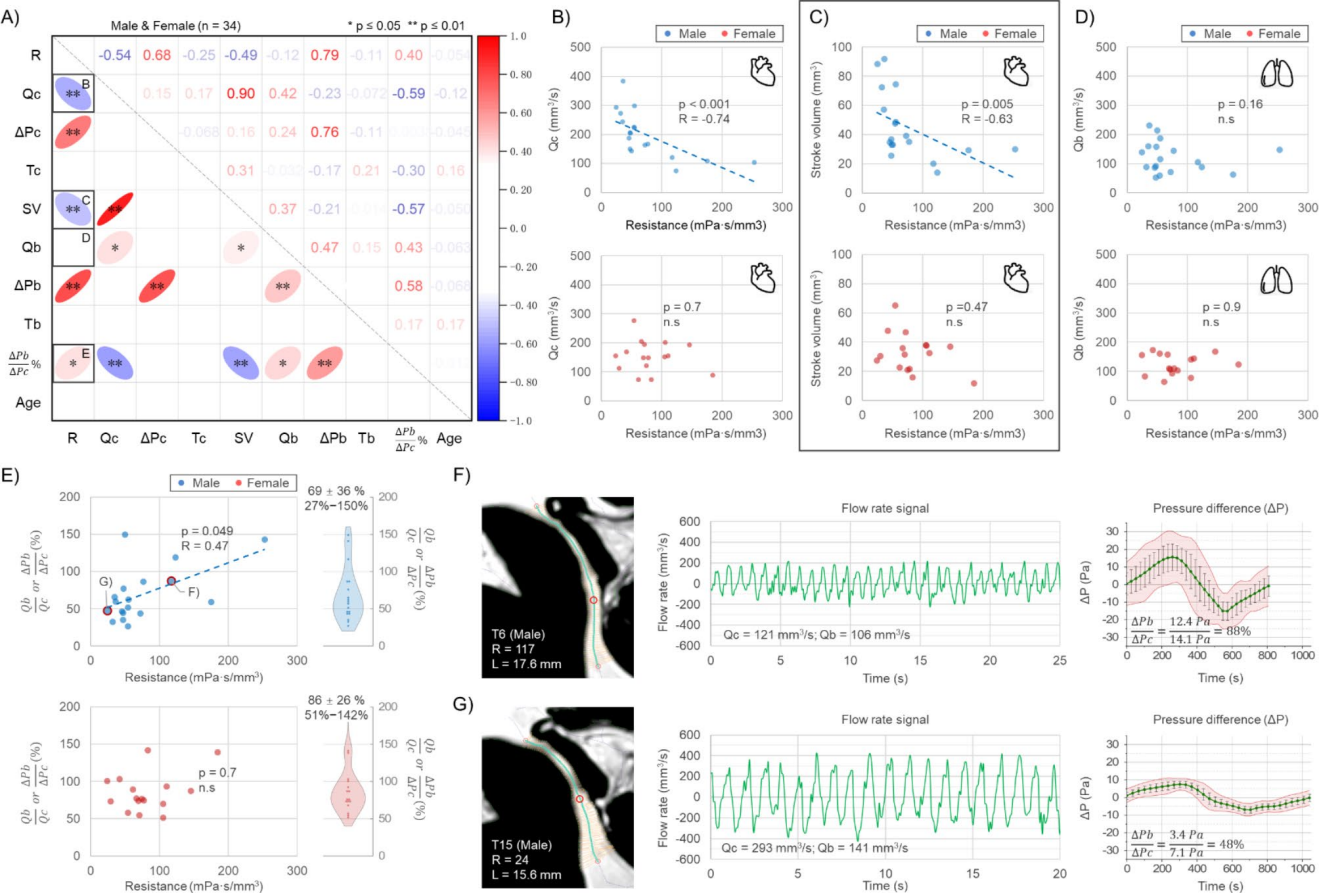
(**A**) The Spearman’s correlation matrix of aqueduct parameters for all 34 participants, where the upper triangle of the matrix represents the R values. Qc and Qb represent cardiac-driven and breath-driven flow rates, respectively; ΔPc and ΔPb denote cardiac-driven and breath-driven pressure differences, respectively; Tc and Tb indicate cardiac and breath period, respectively; SV represents stroke volume, and R stands for resistance. (**B**), (**C**) and (**D**) Scatter plots showing the distribution of resistance with Qc, stroke volume, and Qb, respectively, to further visualize the correlation in males and females. (**E**) Scatter plot showing the distribution of resistance with the percentage of ΔPb/ΔPc in males and females, representing the trend of breath-driven pressure difference relative to cardiac-driven pressure difference as resistance changes. (**F**) and (**G**) demonstrate the morphological images, continuous flow signals and pressure curves for the two male participants labelled in (**E**)

Figure 6-A shows the relationships between aqueduct resistance, flow parameters, and pressure differences, including data from both male and female participants. Resistance (R) is negatively correlated with Qc (*p* < 0.01, *r* = -0.54) but not correlated with Qb (*p* = 0.44, *r* = -0.12). Stroke volume is positively correlated with Qc (*p* < 0.001, *r* = 0.90) and negatively correlated with R (*p* = 0.003, *r* = -0.49). Spearman’s test did not find a significant correlation between stroke volume and cardiac period in this study (*p* = 0.07, *r* = 0.31). Both cardiac-driven and breath-driven ΔP are positively correlated with R, with ΔPb showing a higher correlation with R than ΔPc (*R* = 0.79 vs. *R* = 0.68).

When considering gender, resistance is not correlated with Qc or stroke volume in females (Fig. 6-B&C). Further analysis using a generalized linear model revealed that the influence of gender on the correlation between R and Qc did not reach significance (*p* = 0.075, Fig. S1), nor was a significant effect of gender observed on the correlation between R and SV (*p* = 0.423, Fig. S2).

ΔPb/ΔPc% represents the percentage of breath-driven ΔP relative to cardiac-driven ΔP. Due to the fixed resistance for each participant, ΔPb/ΔPc% equals Qb/Qc%, which can be understood as the percentage of breath-driven flow rate relative to cardiac-driven flow rate. As shown in Fig. 6-E, ΔPb/ΔPc% is higher in females than in males (86% vs. 69%). In males, an increase in resistance corresponds to an upward trend in ΔPb/ΔPc% (*p* < 0.05, *r* = 0.47), but this correlation is not observed in females (*p* = 0.7). In the generalized linear model analysis, the interaction between R and gender did not reach statistical significance (*p* = 0.481, Fig. S3).

Fig. 6-F&G show the morphological segmentation results, the continuous flow rate signals and the ΔP curves of the aqueduct for two male participants. It can be observed that participant T6, who presented higher resistance exhibited significantly smaller Qc and a greater ΔPb/ΔPc%.

## Discussion

The post-processing platform developed in this study for quantifying aqueduct pressure differences effectively balances accuracy and ease of use, providing technical support for clinical measurement of transmantle pressure. This study, for the first time, integrated real-time phase-contrast sequences to quantify aqueduct pressure differences under free-breathing conditions. It also systematically analysed the relationship between aqueduct resistance and flow and pressure differences driven by cardiac and breath activities in both sexes.

### ΔP is not transmantle pressure

It is important to note that transmantle pressure refers to the pressure difference between the ventricular system and the subarachnoid space. The aqueduct pressure difference (ΔP) is not equivalent to transmantle pressure. However, considering that the primary pressure drop between the ventricles and the subarachnoid space occurs at the aqueduct, ΔP should theoretically have a strong correlation with transmantle pressure [17, 25]. On the other hand, direct measurement of transmantle pressure using invasive pressure sensors requires surgical intervention and carries risks of complications. In contrast, non-invasive approaches offer a safer alternative for estimating aqueduct pressure as a representation of transmantle pressure, making them a promising direction for future research and clinical applications.

### Efficient and accurate quantification of aqueduct ΔP

If ΔP is to be used as a clinical diagnostic indicator, it is essential to accurately quantify individual differences in ΔP rather than relying on approximate values. Whether using the Poiseuille equation or the Navier-Stokes equation, the diameter of the aqueduct as a function of its length is a critical parameter. The diameter of the aqueduct varies significantly along its length (Fig. 5A), making finite element segmentation necessary to account for these variations and ensure accurate resistance calculations.

Furthermore, in order to minimise the potential for human error between operators, the post-processing workflow must be standardised, with an emphasis on the utilisation of automated algorithms to reduce the manual intervention. For example, the platform developed in this study employs an automatic threshold definition utilising a line segment during the binarization process. Additionally, the position where 95% of the aqueduct resistance occurs is employed as the effective length (L), thus removing segments that have a minimal effect on resistance.

It is also important to consider the partial volume effect, which arises due to the typically low resolution of phase-contrast sequences, particularly RT-PC [37–39], leads to a significant overestimation of the aqueduct area. Additionally, the low spatial resolution further reduces the ability to quantify inter-individual differences in aqueduct area. As demonstrated in Fig. 3-D, there was no correlation between the aqueduct diameter measured by RT-PC and the resistance. Consequently, it is imperative to utilise a high spatial resolution morphological image to accurately quantify the morphological parameters of the aqueduct. Due to its high SNR and spatial resolution, BFFE is highly suitable for quantifying aqueduct resistance [40, 41]. In this study, a spatial resolution of 0.6 × 0.6 × 1.2 mm³ was used, resulting in only two acquisition pixels at the minimum diameter (~ 1.2 mm). While linear interpolation improved the resolution to 0.03 × 0.03mm^2^, the risk of partial volume effects remains. Future work could address this issue by further improving spatial resolution.

In addition to aqueduct morphology, flow rate also influences the accuracy of ΔP quantification. As previously mentioned, the lower spatial resolution of RT-PC introduces significant partial volume effects, which overestimate the aqueduct cross-sectional area. However, this low spatial resolution also causes an underestimation of the mean velocity within the ROI. Consequently, within a certain spatial resolution range, RT-PC can still achieve flow parameters comparable in accuracy to those measured by conventional cardiac-gated phase contrast, despite the presence of partial volume effects. As a previous study has shown that [28], compared to 1 × 1 mm² conventional phase contrast, 2 × 2 mm² RT-PC overestimates the cross-sectional area by 2.4 times and underestimates the mean velocity by 2.1 times, yet the difference in peak flow remains within 15%, with no significant difference in stroke volume. It is also essential to balance spatial and temporal resolution; as the choice of spatial resolution directly influences temporal resolution, which ultimately affects the accuracy of flow quantification.

The cardiac-driven pressure difference amplitude (twice the ΔPc) obtained in this study (24.2 ± 11.4 Pa, i.e. 0.182 ± 0.086 mmHg) is similar with values quantified by Sincomb et al. [17].

### Aqueduct resistance is a valuable parameter

Although aqueduct resistance is positively correlated with its minimum and average diameter (Fig. 3-D), the interindividual variability of resistance is much greater (Table 1), makes it a more promising clinical diagnostic marker. For example, while current clinical practice for diagnosing normal pressure hydrocephalus primarily relies on clinical presentation and cerebrospinal fluid pressure assessment [42], aqueduct stroke volume has been proposed as a promising diagnostic biomarker [8, 43, 44]. Incorporating aqueduct resistance as an additional biomarker could markedly enhance diagnostic accuracy, allowing for more sensitive detection of cerebrospinal fluid abnormalities.

### Breathing contribution cannot be ignored

Free breathing, as the most common and natural respiratory pattern, is highly representative and more easily implemented in clinical settings. Additionally, the results from studies on free breathing can provide a reliable baseline reference for subsequent research on other respiratory patterns. Therefore, this study focuses on quantifying the changes in aqueduct fluid dynamics and pressure differentials under the influence of free breathing.

Compared with other breathing patterns, free breathing has less impact on cerebral circulation [29, 45–47]. Nevertheless, the results of this study indicate that the peak flow rate driven by free breathing (Qb) still reaches nearly 80% of that driven by the cardiac cycle (Qc).

Another key finding of this study is that aqueduct resistance (range of 24–253 mPa·s/mm³) is negatively correlated with cardiac-driven peak flow (Qc) but shows no correlation with breath-driven peak flow (Qb) (Fig. 6-D). Firstly, this inverse relationship between resistance and cardiac-driven flow may provide insights into the pathogenesis of conditions like normal pressure hydrocephalus. Future work could explore how a decrease in aqueduct resistance intensifies cardiac-driven flow oscillations, potentially causing ventricular enlargement in NPH.

Secondly, the lack of correlation between Qb and aqueduct resistance suggests that low-frequency oscillations, such as those driven by breathing or ultra-low-frequency oscillations generated by cerebral vascular autoregulation [48], are less influenced by resistance. This aligns with theoretical principles: in high-resistance conduits, high-frequency oscillations attenuate more due to viscous damping, reducing their effectiveness in driving flow. In contrast, low-frequency oscillations produce slower pressure variations that are less affected by viscous forces and can sustain flow despite high resistance. This finding may also apply to other high-resistance CSF pathways, such as perivascular spaces and interstitial fluid regions [1, 49–51]. While some studies suggest that cardiac pulsations are the main driving force of CSF motion in these pathways [52–54], mathematical modelling studies propose that cardiac-driven high-frequency oscillations are less effective in driving glymphatic flow due to rapid attenuation in high-resistance CSF pathways [55, 56]. Conversely, it has been hypothesized that slower oscillations—such as low-frequency or ultra-low-frequency oscillations induced by behaviours like breathing, cerebral vascular autoregulation, or sleep—may be more likely to sustain net flow through high-resistance CSF pathways, although direct evidence is currently limited. The results of this study may provide useful references for the physiology of CSF circulation in high-resistance channels and future quantification methods.

Furthermore, a previous study quantified the hydrodynamic changes in the aqueduct under free-breathing conditions, confirming that Qc varies with the breathing cycle [28]. Specifically, Qc reaches its maximum value after breathing drives CSF to flow rapidly out of the cranial cavity (Qb^−^). The relationship between the breath-driven Qb variation and aqueduct resistance deserves further investigation in future studies.

Consequently, whether investigating novel clinical diagnostic methodologies or elucidating cerebral circulation physiology, the contribution of breathing cannot be overlooked.

### Gender differences should be given attention

Morphological analysis indicates that male brains are generally larger by 9–12% compared to female brains [57, 58]. Whether this gender difference extends to the aqueduct remains inconclusive [59–61]. Our findings support the existence of gender differences in aqueduct morphology, primarily showing that the male aqueduct is longer and has a larger average diameter. However, in this study, no significant differences were observed in aqueduct resistance or minimum aqueduct diameter between males and females (Table 1).

Currently, there is no consensus on the sex differences in aqueduct stroke volume. Some studies have confirmed that males have a significantly higher stroke volume compared to females [62, 63], while other studies have found no sex differences in stroke volume [64, 65]. This study found no significant difference in stroke volume between males and females (*p* = 0.14); however, this lack of significance may be due to insufficient statistical power resulting from the small sample size.

An interesting finding, as shown in Table 2, indicates that in females, there is a significant difference between the bidirectional flow rates driven by breathing (Qb^+^ = 135 mm^3^/s > Qb^−^ = 113mm^3^/s, *p* < 0.01, *r* = 0.54). This indicates that, in the female participants of this study, the maximum flow rate into the ventricles driven by breathing is significantly greater than the maximum flow rate out of the ventricles; this difference was not observed in males (*p* = 0.6). One potential explanation for this phenomenon is the observed difference in free breathing patterns between genders [66, 67]. Another possible explanation is sex differences in brain compliance or in ventricular volume. The reasons behind this result require further investigation.

Another important finding is the differential effect of aqueduct resistance on cardiac-driven flow (Qc) between males and females. A negative correlation between resistance and Qc was found only in males, whereas aqueduct resistance did not affect Qc in females (Fig. 6B&C). Further analysis using a generalized linear model, with Qc as the dependent variable, included R as a covariate, gender as a factor, and the R*gender interaction term (Fig. S1). However, the interaction term did not reach the threshold for statistical significance (*p* = 0.075 > α = 0.05). Therefore, we cannot conclude that there is a gender difference in the correlation between R and Qc, but this is worthy of further investigation with a larger sample size in the future. If there is a gender difference in the effect of aqueduct resistance on CSF hydrodynamics or ΔP, it will provide important insights for a better understanding of neurophysiology and the diagnosis of certain neurodegenerative diseases, such as normal pressure hydrocephalus, Chiari malformation.

Collectively, although no significant differences in aqueduct resistance, Qc, Qb, stroke volume and ΔP were found between males and females, the effect of breathing and aqueduct resistance on CSF hydrodynamics within the aqueduct differs between sexes. Therefore, it is necessary to perform sex-specific analyses in future physiological studies and clinical diagnosis.

### Is there a normal range for aqueduct resistance?

The Monro-Kellie hypothesis postulates that the cranial cavity is a closed and rigid structure, with a constant total volume. This volume is constituted by brain tissue, blood, and CSF [68]. Any increase in the volume of one component must be compensated by a decrease in the volume of another in order to maintain constant intracranial pressure. Consequently, in the event of a change in the total cerebral blood volume (CBV), the CSF will move in the opposite direction as a compensatory mechanism to regulate intracranial pressure. Previous studies have also demonstrated that fluctuations in CBV, influenced by cardiac and breathing activity, serve as the primary driving force for CSF movement [69].

As shown in Fig. 4-D, during the cardiac systole, when CBV and intracranial pressure increase, CSF must flow out of the subarachnoid space to maintain intracranial homeostasis. This outflow predominantly occurs through the cervical spine, the aqueduct, and other potential clearance pathways, such as meningeal lymphatics. According to previous studies [70, 71], during the cardiac systole, approximately 90 − 95% of the CSF flows out of the cranial cavity through the cervical spine, and 5 − 10% flows into the ventricles through the aqueduct. Similar to the cardiac-driven CSF oscillations, free breathing can also provide a lower frequency CBV fluctuation that drives CSF [72–75]. This connection facilitates the exchange of substances between the ventricles and the subarachnoid space, which may enhance the clearance of metabolic waste and contribute to the maintenance of optimal brain health.

Consequently, ΔPc and ΔPb can to some extent mitigate fluctuations in ventricular pressure. For instance, during cardiac systole, some of the pressure is dissipated in the aqueduct in order to drive CSF into the ventricles, thereby preventing the rapid transmission of pressure changes from the subarachnoid space to the ventricles. When the resistance of the aqueduct decreases, ΔPc and ΔPb decrease accordingly (Fig. 6-A), allowing pressure changes in the subarachnoid space to be transmitted more directly to the ventricles, resulting in increased fluctuations in ventricular pressure. This is particularly evident in patients with communicating hydrocephalus, where the aqueducts are significantly enlarged [6, 43]. However, it remains unclear whether ventricular enlargement contributes to further enlargement of the aqueduct, thereby perpetuating a vicious cycle [6, 20, 76].

Consequently, the aqueduct must be open to facilitate the connection between the ventricles and the subarachnoid space. However, it is also necessary to ensure that it has sufficient resistance to mitigate fluctuations in ventricular pressure. Does an optimal normal range for aqueduct resistance exist? This is a question worthy of further investigation, as it may provide optimal strategies for certain neurosurgical procedures, such as ventriculostomy, which involve the reconstruction of the ventricular-subarachnoid space pathway [77, 78]. Perhaps considering the resistance of the new channel to maintain it within an appropriate range could prevent excessive pressure changes in the ventricles.

### Limitations and prospects

Several limitations can be addressed and refined in future research. Firstly, the current platform has not yet developed the capability to calculate ΔP based on the Navier-Stokes equations. Although the average Womersley number in this study is only 3.1, indicating that viscous forces dominate, inertial forces are still present. Using the Poiseuille formula alone neglects these inertial forces, leading to an underestimation of ΔPc. Future work could integrate the Navier-Stokes equations into the platform for cases with high Womersley numbers, such as normal pressure hydrocephalus. Moreover, approximating the aqueduct as a series of discrete cross-sections may introduce bias, as Poiseuille’s law assumes an infinitely long, uniform conduit. The aqueduct’s finite length and varying geometry deviate from these idealized conditions, potentially affecting accuracy. While this approach is practical for the current analysis, future work could employ a water phantom to compare the current quantification method with other ΔP estimation formulas [23, 25, 26], thereby identifying the most suitable calculation approach and further enhancing the platform’s quantitative accuracy.

Secondly, although the cross-sectional area of the cerebral aqueduct is often approximated as circular [32], endoscopic studies have indicated that it can sometimes be elliptical, with inter-individual variability and morphological changes under certain neurological conditions [79, 80]. In our study, despite the high in-plane spatial resolution of the BFFE images, the relatively large slice thickness (1.2 mm) posed challenges for accurately quantifying the aqueduct’s cross-sectional shape. Future studies could upgrade the platform to support three-dimensional model quantification and improve three-dimensional spatial resolution by reducing the slice thickness, thereby better quantifying variations in the aqueduct’s cross-sectional morphology, such as the ratio of the long to short axes of the ROI. In addition, we could further investigate cerebral vascular resistance, pressure, and hemodynamic under the influence of breathing, or, by integrating with 4D flow sequences [81–83], provide technical support for research related to distal cerebral microvasculature [84].

Thirdly, the age distribution of the participants was too concentrated (25 ± 4 years), making the study’s data more suitable as reference values for healthy adults rather than for analysing the impact of age on various parameters. In this study, we only found a negative correlation between age and the Ls/L ratio (Fig. 3-D, Fig. S4 & Fig. S5).

Fourthly, it is necessary to assess both inter- and intra-operator consistency when using this platform to quantify aqueduct morphology and ΔP. Such evaluations will verify the reliability of the platform’s quantitative outputs and help identify parameters with relatively low measurement stability. By pinpointing these less stable parameters, targeted approaches—such as repeated measurements—can be employed to further enhance the overall accuracy of the quantified results.

Finally, the limited sample size applied to sex-specific analyses in this study may have resulted in insufficient statistical power, increasing the likelihood of type II errors and potentially leading to inaccurate or unreliable conclusions. Consequently, the results regarding gender-based differences should be interpreted with caution. It is recommended that future studies increase the sample size in order to validate these findings. Additionally, if sex differences in aqueduct fluid dynamics and transmantle pressure do exist, future studies could explore the mediating factors of these differences by incorporating additional physiological parameters such as brain volume, brain compliance, heart rate variability, Waist-to-Hip Ratio, Body Fat Percentage, VO2 Max, etc. These measures may provide deeper insights into the mechanisms behind potential sex-specific variations in cerebrospinal fluid dynamics.

## Conclusion

The post-processing platform developed in this study enables accurate and straightforward quantification of aqueduct resistance and various morphological parameters. By integrating flow data, the platform allows us to calculate the pressure differences across the aqueduct. The highly automated workflow minimizes errors caused by manual operation variability. This platform will provide valuable support for future studies on transmantle pressure and the development of new clinical diagnostic approaches for degenerative diseases.

This study is the first to quantify aqueduct pressure differences driven by cardiac and breathing activities, utilising this platform in combination with real-time phase contrast MRI. Based on the results of this study, we provide a preliminary analysis of the relationships between aqueduct resistance, flow parameters, and pressure differences, including gender-related observations. The study’s key findings suggest that the aqueduct pressure difference driven by free breathing can reach nearly 80% of that driven by cardiac activity, and that changes in aqueduct resistance primarily affect cardiac-driven flow, with no significant impact on breath-driven flow, highlighting the significance of breathing activity. These findings may serve as valuable preliminary references for future studies on the effects of breathing on cerebral circulation, intracranial pressure, and transmantle pressure.

## Electronic supplementary material

Below is the link to the electronic supplementary material.

Supplementary Material 1

## Data Availability

No datasets were generated or analysed during the current study.

## Abbreviations

ΔPc^+^: The maximum cardiac-driven ΔP from the fourth to the third ventricle
ΔPb^−^: The maximum breath-driven ΔP, from the third to the fourth ventricle
ΔPb: The average of ΔPb^+^ and ΔPb ^−^
BFFE: Balanced Fast Field Echo
CBV: Total cerebral blood volume
CSF: Cerebrospinal fluid
D-mean: Average diameter of the aqueduct
D-min: Minimum diameter across aqueduct elements
D-PC: The diameter of the aqueduct measured by RT-PC
L: Path length of the aqueduct
Ls: Straight-line distance from the start to the end point
L-defined: The operator-defined length of the aqueduct
Qc^+^: The maximum cardiac-driven flow rate, from the fourth to the third ventricle
Qc^−^: The maximum cardiac-driven flow rate, from the third to the fourth ventricle
Qc: The average of Qc^+^ and Qc^−^. Half of flow rate amplitude
Qb^+^: The maximum (free) breath-driven flow rate, from the 4th to the 3rd ventricle
RT-PC: Real-time phase-contrast MRI
