# Supplementary material for: Transmantle pressure under the influence of free breathing: non-invasive quantification of the aqueduct pressure gradient in healthy adults
Source: Fluids Barriers CNS. 2025 Jan 3;22:1. doi: 10.1186/s12987-024-00612-x (PMC11697896; doi:10.1186/s12987-024-00612-x)
Supplement: Supplementary file 1 — Supplementary Material 1 [file 12987_2024_612_MOESM1_ESM.docx]

Additional data

# 1. Interaction Analysis of Gender Moderation in the Resistance (R)-Cardiac driven flow rate (Qc) Relationship


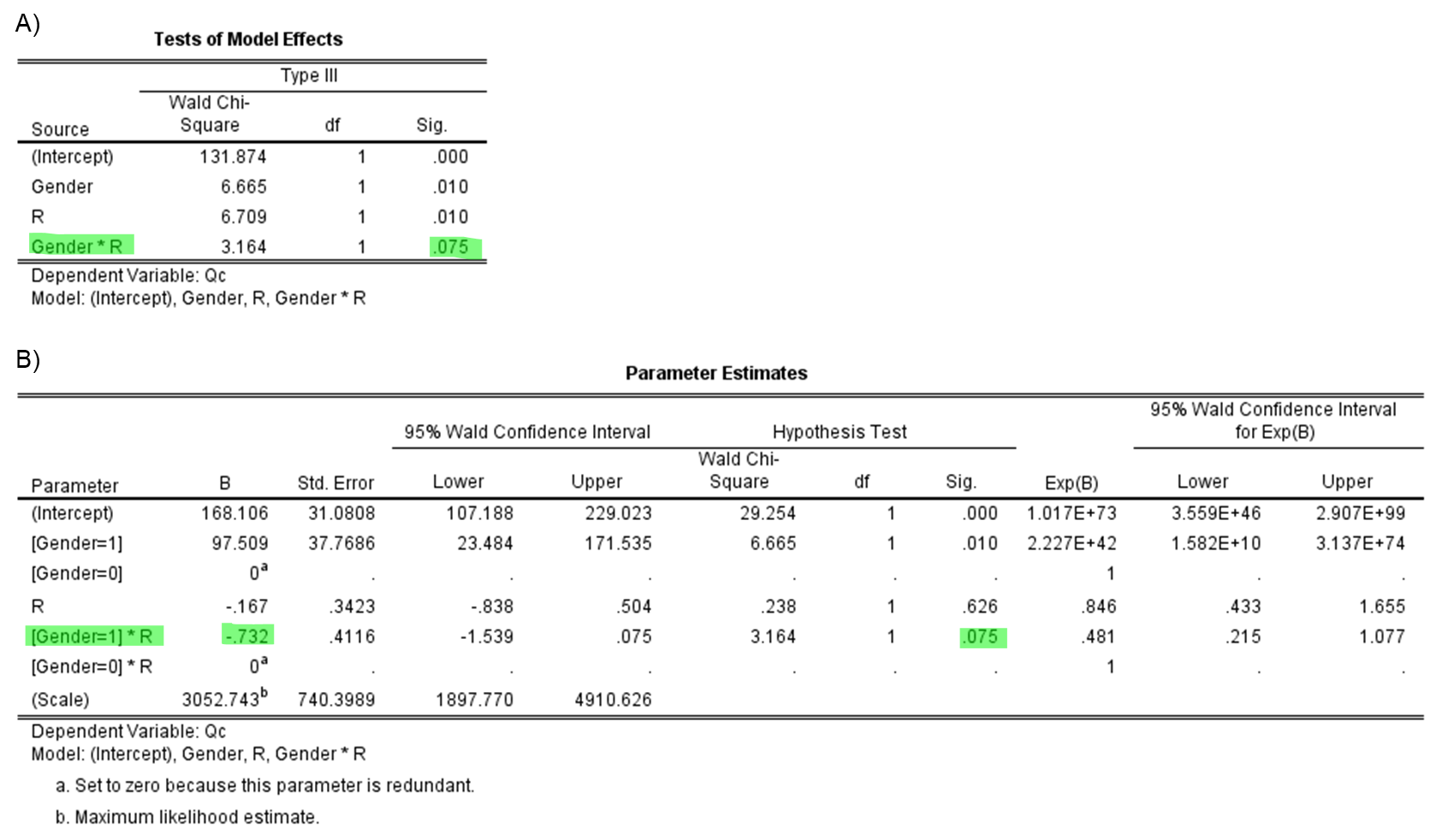


**Fig. S1 Generalized Linear Model (GLM) Analysis of the Effects of R, Gender, and the R × Gender Interaction on Qc.** A) Table of tests of model effects and B) Table of Parameter Estimates.

We conducted a Generalized Linear Model (GLM) analysis to assess whether gender moderates the relationship between R and Qc. In this model, Qc was treated as the dependent variable, gender as a categorical factor (0 = female, 1 = male), and R (resistance) as a continuous covariate. The model was designed to evaluate both the main effects of R and gender on Qc, as well as the interaction effect between R and gender (Gender × R). Specifically, the analysis aimed to determine if the correlation between R and Qc differs significantly between males and females by examining whether gender significantly modifies the strength of the relationship between R and Qc., the following results were obtained:

- Gender had a significant effect on Qc (p = 0.010), indicating a significant difference in Qc between males and females.
- R (Resistance) also showed a significant effect on Qc (p = 0.010), suggesting that changes in R have an impact on Qc.
- The interaction term (Gender × R) was near significance (p = 0.075), indicating **only a potential moderating role of gender in the relationship between R and Qc.** In males, the negative correlation between R and Qc appeared stronger (B = -0.732). However, this interaction did not reach statistical significance, **so** **we cannot definitively conclude that gender significantly influences the R-Qc relationship**. Further studies with a larger sample size may be needed to confirm these findings.

This result suggests a potential trend but does not provide sufficient evidence to claim a statistically significant gender difference in the relationship between R and Qc.

# 2. Interaction Analysis of Gender Moderation in the Resistance (R)-Stroke volume (SV) Relationship


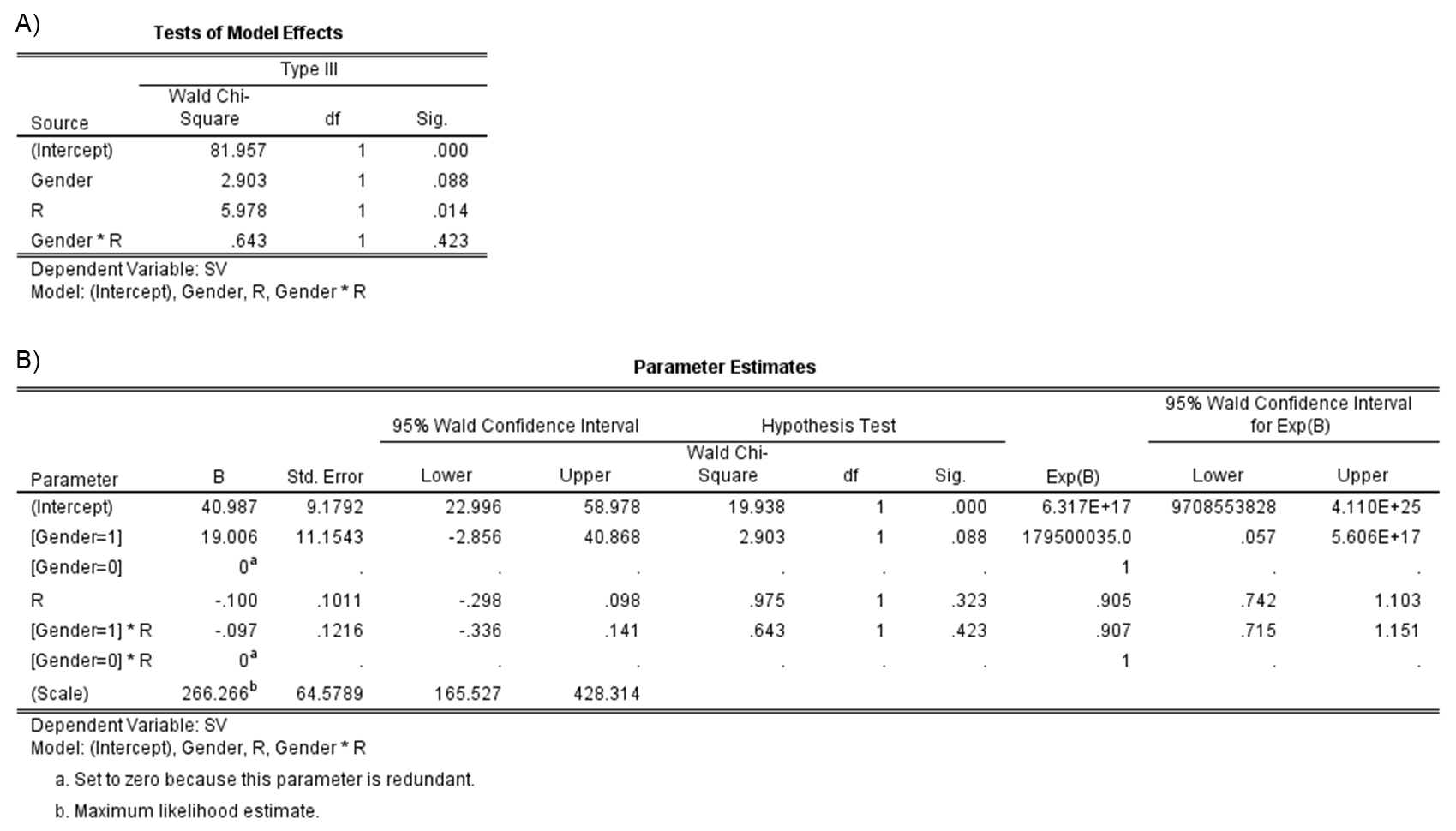


**Fig. S2 Generalized Linear Model (GLM) Analysis of the Effects of R, Gender, and the R × Gender Interaction on** **SV.** A) Table of tests of model effects and B) Table of Parameter Estimates.

We used a Generalized Linear Model (GLM) to further evaluate whether gender significantly affects the relationship between R and SV. In the model, gender was included as a factor, R as a covariate, and SV as the dependent variable. The model included the main effects of gender and R, as well as their interaction (R * Gender). The results showed that R had a significant effect on SV (p = 0.014), and gender had a marginal effect on SV (p = 0.088). However, the interaction between R and gender was not statistically significant (p = 0.423), and therefore, we cannot conclude that gender affects the relationship between R and SV in this study.

# 3. Interaction Analysis of Gender Moderation in the Resistance (R)-Qb/Qc% Relationship


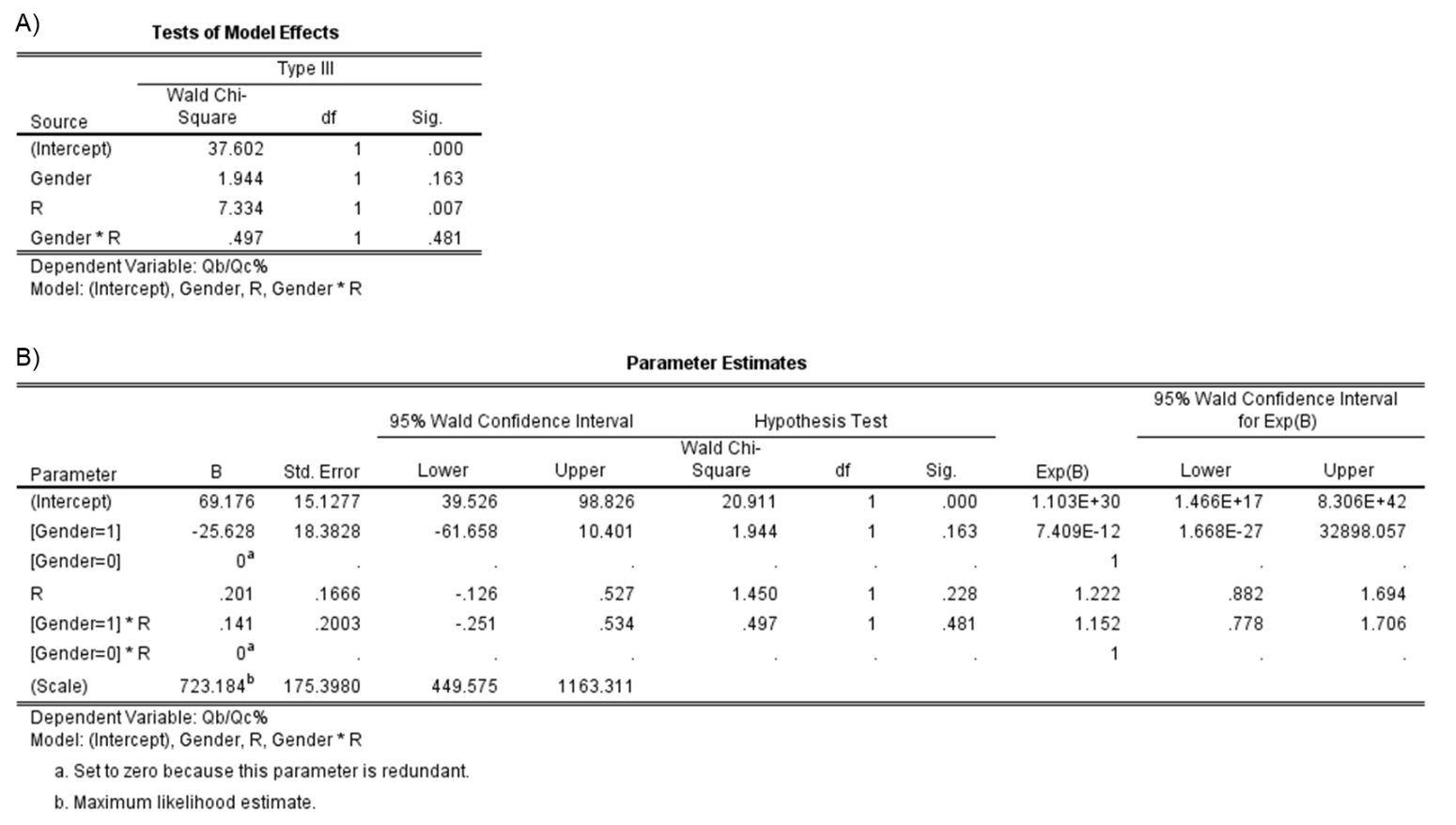


**Fig. S3 Generalized Linear Model (GLM) Analysis of the Effects of R, Gender, and the R × Gender Interaction on** **Qb/Qc%.** A) Table of tests of model effects and B) Table of Parameter Estimates.

We used a Generalized Linear Model (GLM) to evaluate whether gender significantly influences the relationship between R and Qb/Qc%. In the model, gender was set as a factor, R as a covariate, and Qb/Qc% as the dependent variable. The model included the main effects of gender and R, as well as their interaction (R * Gender). The results showed that R had a significant effect on Qb/Qc% (p = 0.007), while the effect of gender on Qb/Qc% was not statistically significant (p = 0.163). Additionally, the interaction between R and gender did not reach statistical significance (p = 0.481), and therefore, we cannot conclude that gender significantly moderates the relationship between R and Qb/Qc%.

# 4. The correlation between Age and L/Ls


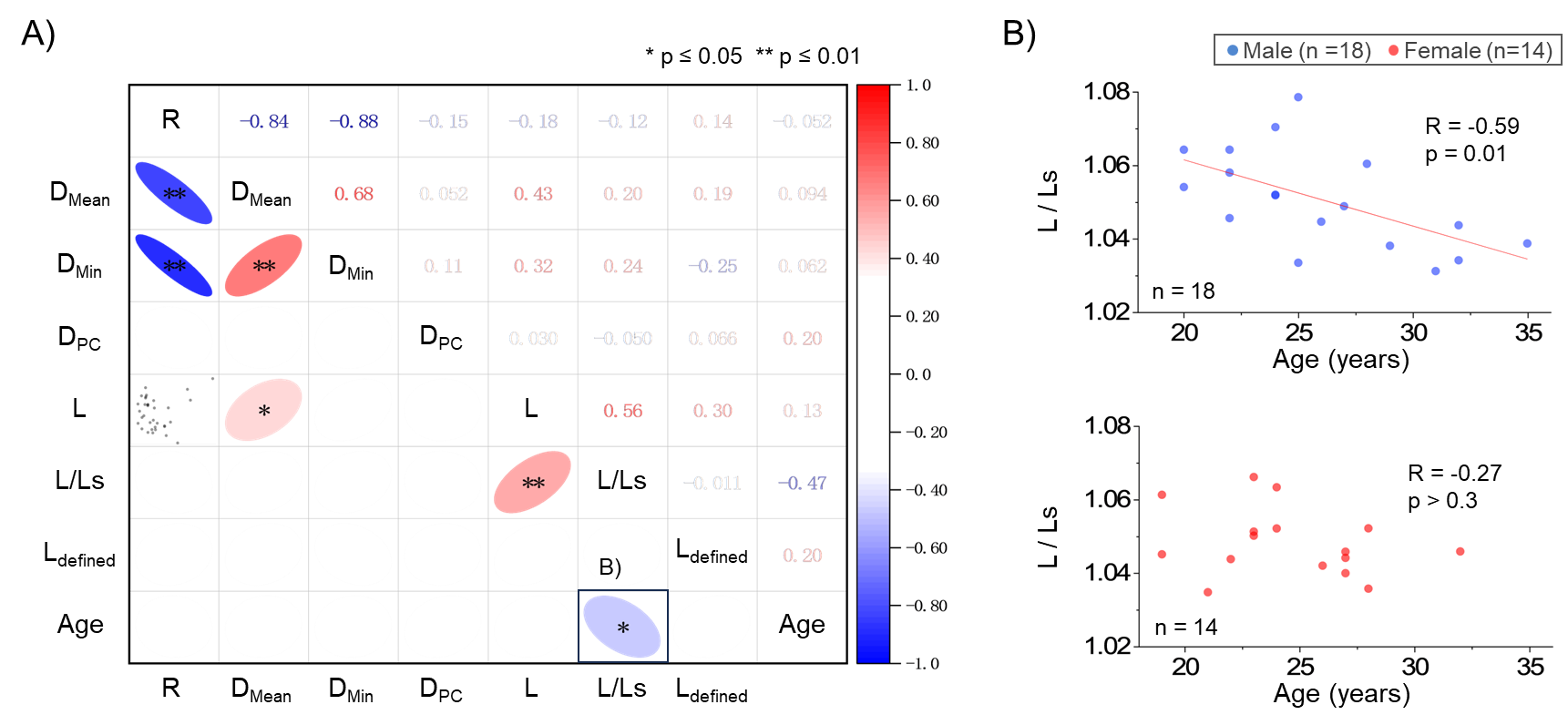


**Fig. S4 Correlation matrix and scatter plots between age and the L/Ls ratio.** A) Correlation matrix of morphological parameters with Spearman's correlation test, the upper triangle of the matrix represents the r values, while the lower triangle shows correlation ellipses and significance levels, with * indicating p < 0.05 and ** indicating p < 0.01; the color bar corresponds to the r values. R for resistance and DPC for diameter measured by RT-PC. B) Scatter plots depicting the relationship between age and the L/Ls ratio.

The ratio of the path length (L) of the aqueduct to its straight-line length (Ls) can, to some extent, reflect its degree of curvature. The straighter the aqueduct, the closer the L/Ls ratio approaches 1. Although the curvature of the aqueduct in this study shows a relatively narrow range (1.03−1.08), this parameter may still influence CSF hydrodynamics and ΔP to some extent. Therefore, it is necessary to perform correlation analyses with other parameters. From Fig. S4-A we can see that age does not correlate with L or Ls but negatively correlates with L/Ls (Fig. S4-A). suggesting that the aqueduct tends to become straighter with increasing age.

Fig. S4-B displays scatter plots of age versus L/Ls, with gender differences highlighted. It can be observed that the age of the females does not correlate with the L/Ls, whereas the L/Ls of the males decrease with increasing age. Further analysis using a generalized linear model, presented in Fig. S5, demonstrated a significant negative association between age and L/Ls (p = 0.019), consistent with the results obtained from the Spearman correlation analysis. However, the interaction term for age and gender was not statistically significant (p = 0.189), suggesting that the effect of age on L/Ls does not significantly differ between males and females.

# 5. Interaction Analysis of Gender Moderation in the Age-L/Ls Relationship


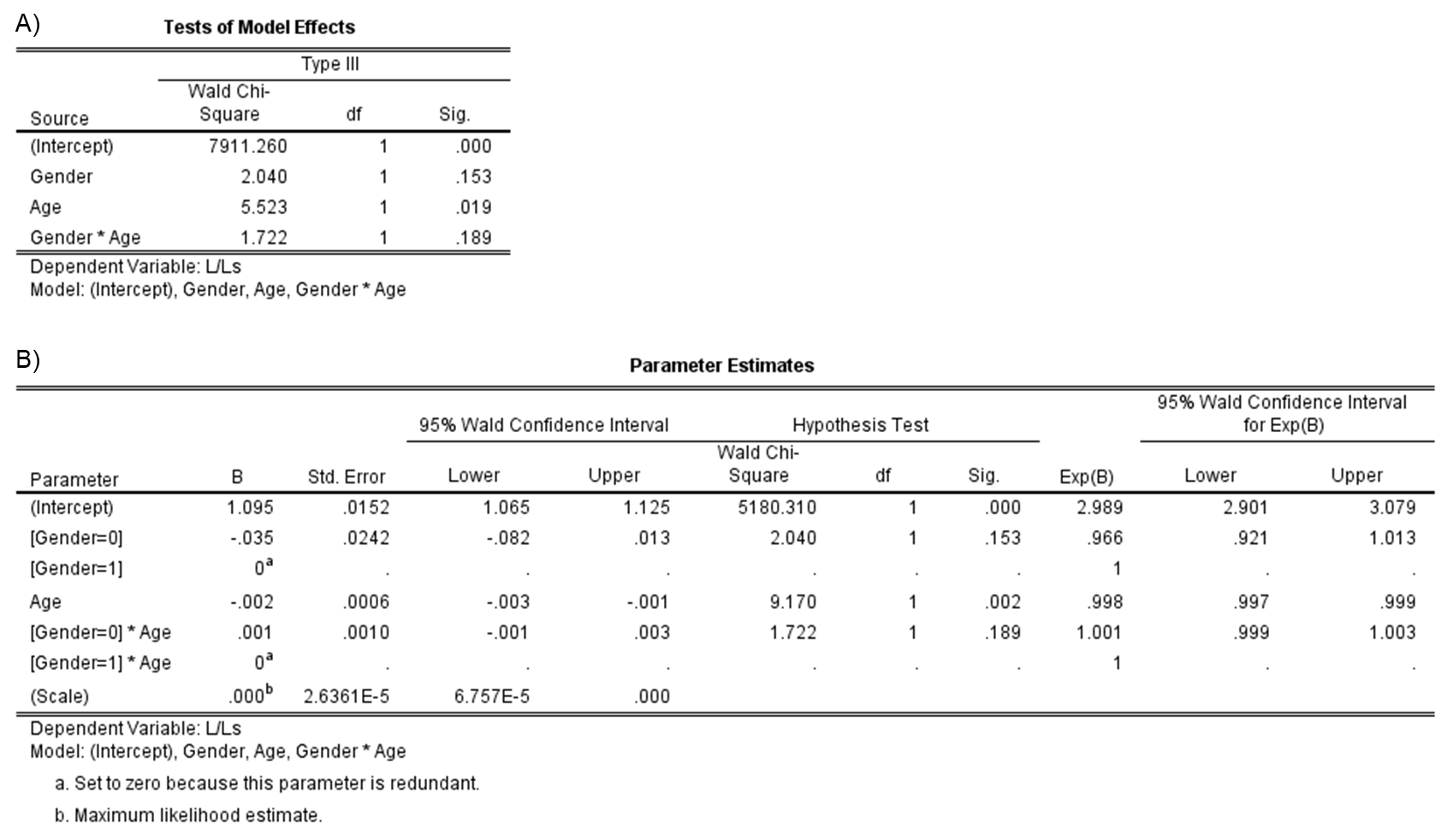


**Fig. S5 Generalized Linear Model (GLM) Analysis of the Effects of age, Gender, and the age × Gender Interaction on L/Ls.** A) Table of tests of model effects and B) Table of Parameter Estimates. L and Ls are the path length and the direct linear distance from the starting point to the endpoint of aqueduct.

We used a Generalized Linear Model to evaluate whether gender significantly influences the relationship between age and L/Ls. In the model, gender was set as a factor, age as a covariate, and L/Ls as the dependent variable. The model included the main effects of gender and age, as well as their interaction (Gender * Age). The results showed that age had a significant effect on L/Ls (p = 0.019), while the effect of gender on L/Ls was not statistically significant (p = 0.153). Additionally, the interaction between age and gender did not reach statistical significance (p = 0.189), and therefore, we cannot conclude that gender significantly moderates the relationship between age and L/Ls.

# 6. Reconstruction Methods for Cardiac-Driven and Breath-Driven Flow


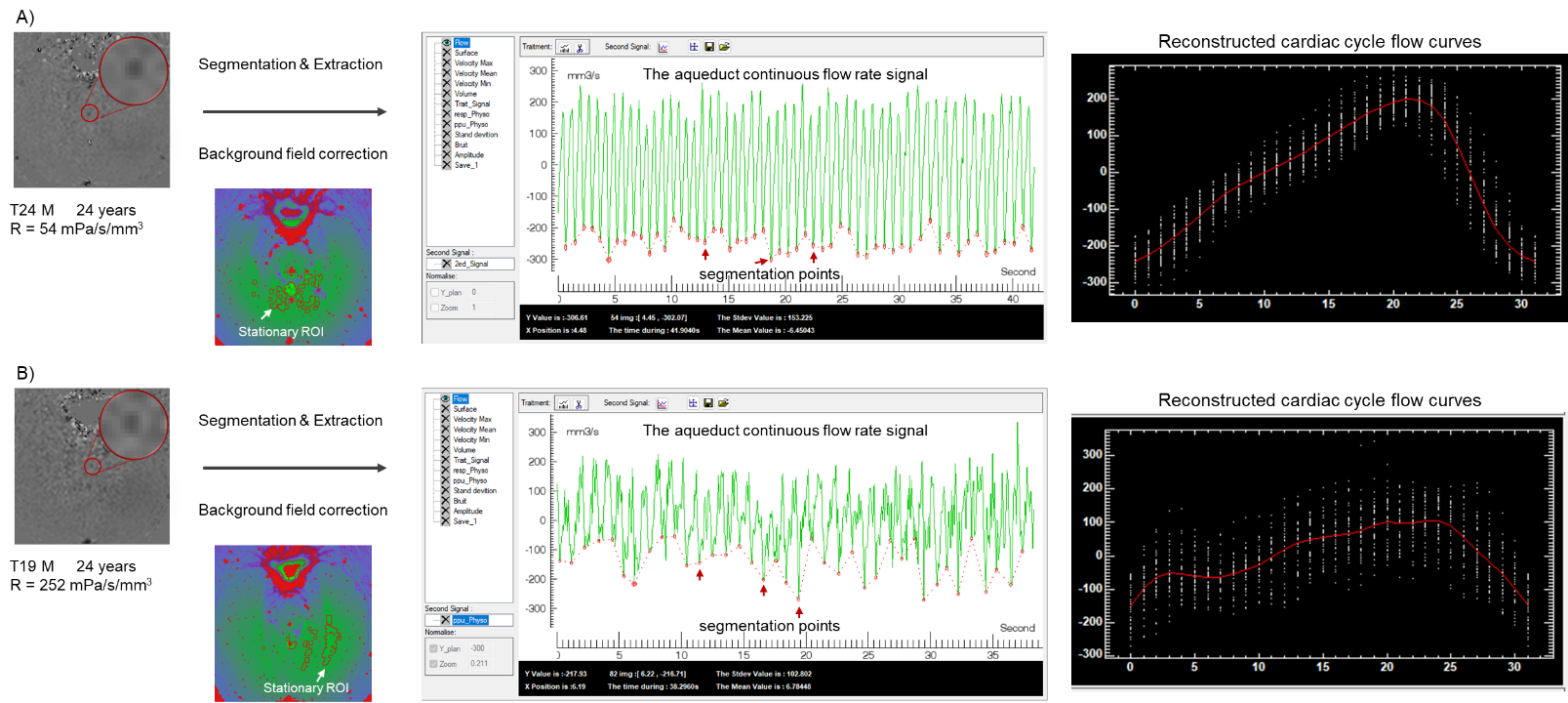


**Fig. S6 The extraction process for continuous aqueduct flow rate signals in T24 A) and T19 B).**

The continuous aqueduct flow signals are obtained through segmentation and background correction. T28 in Fig. S5-A represents a participant with low resistance (R = 54 mPa·s/mm³), and T27 in Fig. S5-B represents a participant with high resistance (R = 252 mPa·s/mm³). Using automated function, the lowest point in each cardiac cycle is identified for segmentation. These cycles are interpolated into 32 sampling points and reconstructed into an average cardiac cycle flow curve. The white dots show the distribution of these cardiac cycles. When respiratory effects are more prominent, greater variability in the white dots is observed, reflecting changes in both net flow and amplitude due to respiration.

Compared to traditional frequency-domain methods, this approach offers several advantages:

1. **No need for respiratory frequency input:** Frequency-domain analysis typically requires respiratory signals to determine the exact frequency range for accurate filtering. Our method, however, segments the continuous signal in the time domain without requiring prior knowledge of respiratory frequency, simplifying data acquisition and processing, especially when paired with automatic software.
2. **Accounts for multiple flow parameters affected by respiration:** Traditional bandpass filtering isolates the low-frequency component to represent respiration. However, studies show that respiratory also impact other parameters, such as the flow rate amplitude of the cardiac cycle. By using the 95% limits of agreement (LOA) from the reconstructed signal, this method captures changes in multiple variables beyond just net flow rate.
3. **More convenient and robust:** This method automatically averages Qc and Qb over multiple cardiac cycles, eliminating the need for additional signal analysis. By setting LOA confidence bands, outliers can be excluded also.

Of course, the method has its limitations, which may mistakenly attribute ultra-low frequencies effects to respiration. This issue can be mitigated by applying low-pass filters to remove components below 0.1 Hz.
